# Fisher Information and the Dynamics of Multicellular Ageing

**DOI:** 10.3390/e27060638

**Published:** 2025-06-15

**Authors:** Zachary F. Hale, Gonzalo A. Cánez, Thomas C. T. Michaels

**Affiliations:** 1 Department of Biology, Institute of Biochemistry, ETH Zurich, Otto Stern Weg 3, 8093 Zürich, Switzerland; 2 Bringing Materials to Life Initiative, ETH Zurich, 8093 Zürich, Switzerland

**Keywords:** somatic evolution, senescence, cancer, ageing, Fisher information

## Abstract

Information theory has long been integrated into the study of biological ageing, for example, in examining the roles of genetic and epigenetic fidelity in cellular and organismal longevity. Here, we introduce a theoretical model that interprets ageing in multicellular systems through the lens of Fisher information. Previous theories have suggested that the ageing of multicellular organisms is an inevitable consequence of the inherent tension between individual cell reproduction and the homeostasis of the multicellular system. Utilising concepts from information theory and statistical mechanics, we show that Fisher information parametrises the dynamics of this tension through non-monotonic behaviour, which depends on an optimal balance of competition and cooperation between cells. Moreover, Fisher information suggests that the ability to infer true biological age from a sample evolves through complex dynamics over an organism’s lifespan.

## 1. Introduction

Multicellular life faces a fundamental conflict between individual cell competition and intercellular cooperation, whereby cells devote some degree of their individual resources to the common good [1,2]. Cooperative strategies can be summarised in five groups which benefit the whole but at a cost to individual cells: inhibition of cell proliferation, transport of resources, programmed cell death, division of labour, and creation and maintenance of the extracellular environment [2,3,4,5,6,7]. These processes are necessary and advantageous to multicellular life, requiring that cells sacrifice some measure of resources to promote the functioning and maintenance of the organism. However, cooperation between cells creates networks of interdependence characterised by shared resources and information, which in turn engenders potential for damage accumulation and ageing. Previous work has shown that interdependence within generalised networks of failure-prone components leads to characteristic ageing dynamics and, eventually, system collapse in both living and engineered systems [8,9,10,11,12,13]. An ubiquitous solution to mitigate ageing-induced degradation involves renewal and repair. In multicellular organisms, one such type of maintenance involves controlled cell proliferation. This mechanism, however, opens opportunities for deleterious somatic mutations, which can lead to a gradual deterioration of cellular functions and ultimately culminate in cellular senescence [14,15,16]. Senescent cell accumulation can be harmful to the vitality of the organism and has been linked with various age-related disorders such as cardiovascular and neurodegenerative diseases [16,17].

Competition between cells offers a mechanism to eliminate unfit senescent cells, potentially extending organismal vitality [2,16,17,18,19,20,21,22]. However, excessive intercellular competition can promote the proliferation of non-cooperative, “cheater” cells, such as cancer cells, which ultimately undermines organismal vitality [1,2,23,24,25,26,27]. These “cheater” cells enjoy a selective advantage over cooperative cells by eschewing investments in cooperative traits that benefit the multicellular organism but reduce their individual fitness [19,28]. Given these dynamics, the emergence of such cheaters and the consequent decline in cellular cooperation appear inescapable [14,29,30,31,32].

## 2. Ageing as Optimal Competition

A recent study [14] explores cancer and senescence as inevitable consequences of the dilemma of competition, and acts as our conceptual basis for modelling ageing. Essentially, competition is a double-bind where either cancer or senescence is favoured depending on the strength of competition. Organisms have therefore evolved an optimal level of competition which eliminates senescent cells but delays the onset of cancer, temporarily prolonging overall vitality. This interplay is a general model of ageing which relates competition, cancer, and ageing, where ageing is an intrinsic and inevitable characteristic of multicellular life. It is also compatible with existing explanations where ageing is a failure to select alleles which preserve the organism in late life; however, in this case, ageing is inevitable even if selection is perfect [14].

In our previous work [33], we introduced a minimal analytic framework using master equations to encapsulate these dynamics of multicellular ageing, where ageing is a problem of optimal competition. This is essentially a toy model which expresses key dynamics in the problem of optimal competition. Here, we show that this model can be reformulated in Lagrangian form. The resulting Lagrangian corresponds to Fisher information, thus providing an interpretation of this model of multicellular ageing in terms of information theory. Specifically, we introduce Fisher information to the study of ageing-as-optimal-competition, introducing a novel kind of information in ageing, to which biological relevance may be attributed.

## 3. Master Equation for the Dynamics of Multicellular Ageing

**Figure 1:**
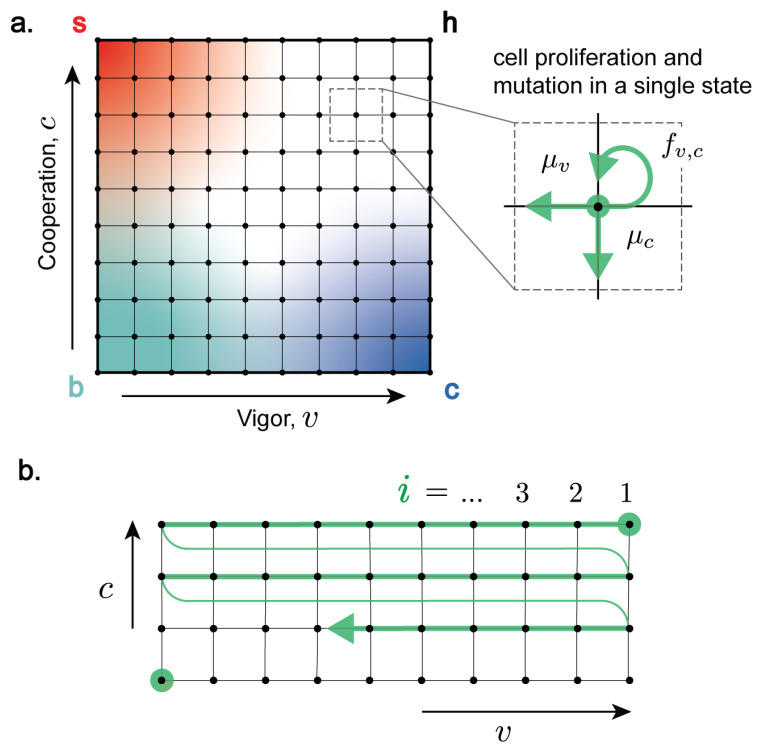
(**a**) Cell vigour *v* and cooperation *c* define a two-dimensional coordinate system (v,c) of cell states. Progressive loss of vigour corresponds to cell senescence, while progressive loss of cooperation corresponds to cancer. The dynamics of the population $\rho_{v,c}\left(t\right)$ of cells of type (v,c) at time *t* are described in terms of transition rates between different states. **(Inset)** Populations $\rho_{v,c}\left(t\right)$: (i) proliferate with state-dependent rate $f_{v,c}$ and (ii) mutate (represented by transitions that lower vigour (v→v−1, with rate $\mu_{v}$)) or cooperation (c→c−1, with rate $\mu_{c}$). (**b**) Enumeration of cell states using a single index *i* starting from top right (healthy cells) and moving row-by-row in the direction of decreasing vigour and senescence, terminating at (v=0,c=0).

We consider a multicellular organism with cells characterised by two traits based on the notion that cells use resources to maintain their own fitness, but also sacrifice resources to cooperate: vigour *v* and cooperation *c* [14,33]. We use vigour *v* as a general measure of the resources available to the cell or cellular function. Healthy cells have high vigour, while senescent cells have low vigour. Cooperation *c* measures the fraction of resources that a cell devotes to cooperative activities necessary for the functioning of the organism (division of labour, resource allocation, creation and maintenance of the extracellular environment, proliferation inhibition and controlled cell death, etc. [1,2]). Healthy cells are highly cooperative, while cancer cells have low cooperation. We assume that vigour and cooperation take discrete values 0≤v≤m and 0≤c≤n, defining a two-dimensional coordinate system of cell types (v,c) (Figure 1). On this coordinate system, “healthy” cells (“h”) are highly functioning and cooperative, thus have high vigour (v=m) and high cooperation (c=n). Cooperative cells with low vigour are “senescent” (“s”), while vigorous cells with low cooperation are “cancerous” (“c”). Cells with low vigour and cooperation are classified as both senescent and cancerous (“b”).

We describe the system dynamics on this two-dimensional coordinate system as a the result of (i) cell proliferation (via intercellular competition) with replication rate $f_{v,c}$, which depends on state (v,c) according to Equation (3), and (ii) somatic mutations that affect vigour or cooperation. We assume that mutations change either *v* or *c* in single steps with rates $\mu_{v}$ and $\mu_{c}$ for senescence- and cancer-causing mutations, respectively, as in our previous work, where we found that results from a continuous model were not qualitatively different [33]. Moreover, mutations in *v* and *c* are independent from each other, and we do not account for complex genetic interactions, e.g., epistatic interactions.

We use a master equation to describe the time evolution of the fraction $\rho_{v,c}\left(t\right)$ of cells in state (v,c) at time *t* [33]:

(1) $$\begin{array}{cc} \frac{𝜕\rho_{v,c}\left(t\right)}{𝜕t}= & \underset{\mathrm{selection}\;(\mathrm{competition})}{\underset{︸}{\left(f_{v,c}-\langle f\rangle \right)\;\rho_{v,c}\left(t\right)}}\\ + & \underset{mutations\;in\;v\;space}{\underset{︸}{\mu_{v}\;\rho_{v+1,c}\left(t\right)-\mu_{v}\;\rho_{v,c}\left(t\right)}}+\underset{mutations\;in\;c\;space}{\underset{︸}{\mu_{c}\;\rho_{v,c+1}\left(t\right)-\mu_{c}\;\rho_{v,c}\left(t\right)}}\; \end{array}$$

where

(2) $$\langle f\rangle =\sum_{v,c}f_{v,c}\;\rho_{v,c}\left(t\right)$$

is the population averaged competition. The terms on the first line of Equation (1) describe the effect of selection via intercellular competition: the fraction of cells with $f_{v,c}$ larger than the average value 〈f〉 will increase over time, while cells with $f_{v,c}<\langle f\rangle$ will be purged away by selection. The terms on the second and third lines of Equation (1) describe the effect of somatic mutations that lower vigour and cooperation, respectively, with mutation rates $\mu_{v}$ and $\mu_{c}$. We choose $\mu_{v}$ values to be much greater than $\mu_{c}$, reflecting the fact that only about one percent of human genes are linked to cancer risk [14].

To close the system of equations, we need to specify a relationship for the replication rate $f_{v,c}$ as a function of (v,c) which reflects the competitive strength of different phenotypes. We choose a linear fitness function

(3) $$\frac{f_{v,c}}{f_{0}}=\left(1-k\right)\;\frac{v}{n}+k\;\left(1-\frac{c}{m}\right)\;,$$

where *k* is the cost of cooperation and $f_{0}$ is the highest fitness. With this choice, cancer cells with v=m and c=0 have the highest fitness, $f_{\mathrm{cancer}}=f_{0}$. Senescent cells with v=0 and c=m have zero replication rate, $f_{\mathrm{senescent}}=0$, while healthy cells with v=n and c=m have intermediate fitness, $f_{\mathrm{healthy}}=f_{0}\left(1-k\right)$. This choice effectively models the selective advantage of cells that either have access to high levels of resources (high vigour) or minimise resource investment in cooperative functions (low cooperation), reflecting the selection pressure due to competition that favours less cooperative “cheater” cells. The fitness landscape is therefore skewed towards cancerous phenotypes, highlighting that cheating provides a benefit in competition.

### Time Evolution of Cell Fractions and the Inevitability of Multicellular Ageing

The population dynamic model Equation (1) describes multicellular ageing as an inevitable consequence of the trade-offs between cell competition and cooperation, where the selective pressures for individual cell proliferation conflict with those for maintaining organismal vitality, an idea discussed in [14]. Figure 1a shows an example of the time evolution of cell fractions for a four-state model, where n=m=1. In this case, cells can be in one of four states: healthy (‘h’), senescent (‘s’), cancerous (‘c’), and both senescent and cancerous (‘b’). The dynamics of the four-state cell model unfold in two distinct phases due to the separation of timescales between mutation rates affecting senescence and cancer development. Initially, we observe a first phase where senescent cells accumulate, driven by senescence-causing mutations. Subsequently, the system transitions into a slower second phase, where senescent cells are incrementally eliminated due to the onset of cancer-causing mutations and the selection pressures from non-cooperative cancer cells. As a result, the fraction of cancerous cells begins to increase, displaying a sigmoidal growth curve that eventually plateaus. At the end, healthy and senescent cells always tend to zero, leaving a steady-state of only cells in states c and b.

## 4. Lagrangian Formulation of Master Equation

The master Equation (1) takes the form of a generalised replicator equation with mutation terms that change cell state. We now demonstrate that this equation can be derived from a Lagrangian function that admits an interpretation in terms of the Fisher information.

For this discussion, it is convenient to label two-dimensional cell states (v,c) using a single index *i* in a (n+1)(m+1)-long state vector with components $\rho_{i}$ (Figure 1b). Using this notation, Equation (1) can be written as

(4) $$\begin{array}{cc} \frac{d\rho_{i}}{dt}= & \underset{selection}{\underset{︸}{\left(f_{i}-\langle f\rangle \right)\;\rho_{i}}}+\underset{mutations}{\underset{︸}{\sum_{j}\epsilon_{ij}\;\rho_{j}-\rho_{i}}} \end{array}$$

where $\epsilon_{ij}$ are the entries of a (n+1)(m+1)×(n+1)(m+1) stochastic matrix ϵ with

(5) $$\sum_{i}\epsilon_{ij}=1\;.$$

The off-diagonal terms in ϵ describe mutation transitions from state *j* to state *i*, whereas the diagonal terms account for conservation of probability (Equation (5)). For example, for the four-state model, ϵ is a 4×4-matrix with entries

(6) $$\epsilon =\left[\begin{array}{cccc} 1-\mu_{v}-\mu_{c} & 0 & 0 & 0\\ \mu_{v} & 1-\mu_{c} & 0 & 0\\ \mu_{c} & 0 & 1-\mu_{v} & 0\\ 0 & \mu_{c} & \mu_{v} & 1 \end{array}\right]\;.$$

Populations are ordered h, s, c, b (Figure 1b), such that $\epsilon_{21}$ is the mutation rate from h to s, etc.

In order to derive the Lagrangian form of Equation (1), we introduce a composite proliferation rate $r_{i}$ combining the effect of intercellular competition $f_{i}$ and mutations $\epsilon_{ij}$ as [34,35]

(7) $$\begin{array}{cc} r_{i}= & f_{i}+\sum_{j}\epsilon_{ij}\;\frac{\rho_{j}}{\rho_{i}}\;. \end{array}$$

This transformation allows us to rewrite Equation (1) as an effective replicator equation with replication rate $r_{i}$ (see Appendix A):

(8) $$\begin{array}{cc} \frac{d\rho_{i}}{dt}= & \left(r_{i}-\langle r\rangle \right)\;\rho_{i}\;. \end{array}$$

This result is consistent with previous work that showed that any dynamical system on the N−1 dimensional probability simplex

(9) $$\Sigma^{N-1}=\left\{\rho \in R^{N}\;\left|\;0\leq \rho_{i}\leq 1,\;\sum_{i}\rho_{i}=1\right.\right\}$$

can be formulated as replicator dynamics [36,37]. Using this transformation and after some algebra (see Appendix B), we can reformulate Equation (8) as the Euler–Lagrange equation for the following Lagrangian [34]:

(10) $$\begin{array}{c} L=\sum_{i}\left(\frac{{\dot{\rho_{i}}}^{2}}{\rho_{i}}+\rho_{i}\;{\left(r_{i}-\langle r\rangle \right)}^{2}\right) \end{array}$$

subject to the constraint $\sum_{i}\rho_{i}=1$ (conservation of probability). This Lagrangian takes the classical form L=T−V, where

(11) $$\begin{array}{cc} T & =\sum_{i}\frac{{\dot{\rho_{i}}}^{2}}{\rho_{i}} \end{array}$$

is a kinetic energy term and

(12) $$\begin{array}{cc} V & =-\sum_{i}\rho_{i}\;{\left(r_{i}-\langle r\rangle \right)}^{2} \end{array}$$

is a potential energy term.

The kinetic energy term *T* admits an interesting geometric interpretation. It can be written as

(13) $$\begin{array}{c} T=\sum_{i}\frac{{\dot{\rho_{i}}}^{2}}{\rho_{i}}=\left|\right|\dot{\rho }{\left|\right|}_{\mathrm{FRS}}^{2} \end{array}$$

where

(14) $${\left||X|\right|}_{\mathrm{FRS}}^{2}=\sum_{i}\frac{X_{i}^{2}}{\rho_{i}}$$

is the length of a vector $X\in R^{N}$ in the Fisher–Rao metric, also known as the Fisher information metric, a fundamental concept in information geometry used to measure distances on the probability simplex $\Sigma^{N-1}$ [38,39]. Moreover, information rate takes the distance, i.e., the relative entropy, between two sequential states of a time-dependent p.d.f., effectively measuring the rate of change of the p.d.f. in a path-dependent manner. The information rate τ measures how quickly new information is revealed as a p.d.f. evolves in time [40], and it can be written as

(15) $$\begin{array}{cc} \Gamma^{2} & =\lim_{dt\to 0}\frac{2}{{\left(dt\right)}^{2}}J\left[p\left(x,t+dt\right)|p\left(x,t\right)\right] \end{array}$$

(16) $$\begin{array}{cc} \; & =\int dxp\left(x,t\right){\left({𝜕}_{t}\ln p\left(x,t\right)\right)}^{2} \end{array}$$

where *J* is the Jensen divergence. For more detail, refer to [40].

The dimension of $\Gamma^{2}$ is ${\left(\mathrm{time}\right)}^{-2}$, so, by using units where the length is dimensionless, the information rate can be seen as the kinetic energy per unit mass. Furthermore, $\Gamma^{2}$ can be seen as the Fisher information if time is interpreted as an external control parameter [40]. In this context, information is revealed as cell populations evolve on a characteristic timescale $\Gamma^{-1}\left(t\right)=\tau \left(t\right)$ [40].

Similarly, the potential energy term can be written as

(17) $$\begin{array}{cc} V & =-\langle {\left(r_{i}-\langle r\rangle \right)}^{2}\rangle =\langle r^{2}\rangle +{\langle r\rangle }^{2}=-\mathrm{Var}\left(r\right)\;, \end{array}$$

where

(18) $$\langle X\rangle =\sum_{i}\rho_{i}\;X_{i}$$

denotes the average of the vector $X\in R^{N}$ over the probability distribution $\rho_{i}$. The potential energy *V* therefore measures the variance of the combined replication rate $r_{i}$ over the cell state distribution $\rho_{i}$.

## 5. Link to Fisher Information

We now discuss the link between the Lagrangian Equation (10) and Fisher information. As well as information geometry, Fisher information is a key concept in statistical estimation theory, quantifying the amount of information that an observable random variable carries about an unknown parameter upon which the probability distribution of the random variable depends [38,39]. For a parametric model where the probability distribution $p_{i}\left(\lambda \right)$ of states i=1,2,⋯ varies smoothly with a control parameter λ, the Fisher information is defined as

(19) $$\begin{array}{c} I\left(\lambda \right)=\sum_{i}p_{i}\left(\lambda \right)\;{\left(\frac{𝜕\log p_{i}\left(\lambda \right)}{𝜕\lambda }\right)}^{2}=\langle {\left(\frac{𝜕\log p_{i}\left(\lambda \right)}{𝜕\lambda }\right)}^{2}\rangle \;, \end{array}$$

where $\langle X\rangle =\sum_{i}p_{i}\;X_{i}$ is the average of *X* over the probability distribution $p_{i}$.

Interestingly, the kinetic energy term *T* (Equation (11)) corresponds to the Fisher information associated with the cell state distribution $\rho_{i}$ and the control parameter λ representing time λ=t:

(20) $$\begin{array}{c} T=\sum_{i}\frac{{\dot{\rho_{i}}}^{2}}{\rho_{i}}=\sum_{i}\rho_{i}{\left(\frac{\dot{\rho_{i}}}{\rho_{i}}\right)}^{2}=\sum_{i}\rho_{i}{\left(\frac{𝜕\log\rho_{i}}{𝜕t}\right)}^{2}=\langle {\left(\frac{𝜕\log\rho_{i}}{𝜕t}\right)}^{2}\rangle =I\;. \end{array}$$

Similarly, using the master equation ${\dot{\rho }}_{i}=\left(r_{i}-\langle r\rangle \right)\rho_{i}$, we can rewrite the potential energy term (Equation (12)) as

(21) $$\begin{array}{cc} V & =-\sum_{i}\rho_{i}\;{\left(r_{i}-\langle r\rangle \right)}^{2}=-\sum_{i}\rho_{i}\;{\left(\frac{\dot{\rho_{i}}}{\rho_{i}}\right)}^{2}=-\langle {\left(\frac{𝜕\log\rho_{i}}{𝜕t}\right)}^{2}\rangle =-I\;. \end{array}$$

**Figure 2:**
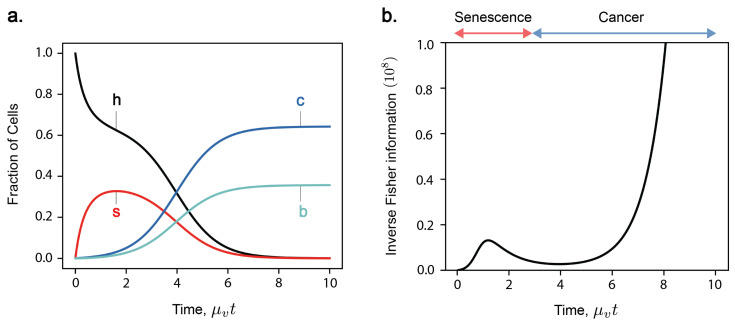
(**a**) Time evolution of the 4-state model (n=m=1) comprising only healthy (‘h’), senescent (‘s’), cancerous (‘c’), and both senescent and cancerous (‘b’) states. The master Equation (1) is solved for k=0.3, $\mu_{v}=10^{-3}$, $\mu_{c}=10^{-5}$, $f_{0}=0.004$ and features a transition from senescence- to cancer-domination. (**b**) Inverse Fisher information 1/I(t) in the four-state model, or the minimum variance in an unbiased estimation of age $t_{biol}$. Inverse Fisher information 1/I(t) exhibits bi-phasic behaviour; rising to a transient maximum in senescence-domination, then rising asymptotically with cancer-domination as a steady-state is reached.

**Figure 3:**
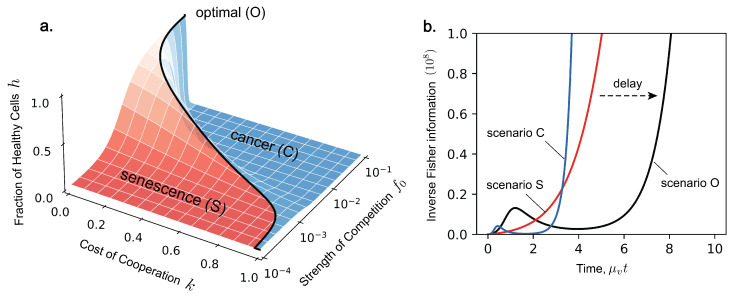
(**a**) The fraction of healthy cells *h* at observation time $t=3\mu_{v}^{-1}$ plotted against the cost of cooperation *k* and the strength of cellular competition $f_{0}$. The solid line indicates the position of the optimum line $f_{0}\left(k\right)$, which maximises vitality. This plot is obtained using the analytical solution for *h* (see [33]) and the same parameters for $\mu_{v}$ and $\mu_{c}$ as in Figure 2. (**b**) The evolution of inverse Fisher information 1/I(t) over the lifespan of the organism for scenario O (k=0.3, $f_{0}=0.004$), scenario S (k=0.25, $f_{0}=0.0001$), and scenario C (k=0.35, $f_{0}=0.01$). Infinite inverse Fisher information corresponds to when cell demographics reach equilibrium, an inevitable outcome delayed by selecting the optimal level of competition.

The Lagrangian Equation (10) is therefore recognised as twice the Fisher information (Figure 2b):

(22) L=T−V=2I

such that Fisher information is a representation of model dynamics. Fisher information is therefore a representation of optimal competition. In the problem of ageing, organisms have evolved an optimal level of competition which prolongs vitality. Depending on the cost of cooperation *k*, there is an optimal strength of competition $f_{0}\left(k\right)$, which maximises organismal vitality at that time, denoted as scenario O. This shown by the black line in Figure 3a at time $t=3\mu_{v}^{-1}$. If cooperation is more costly, then the organism cannot tolerate higher levels of competition. The region towards higher competition is dominated by cancer, denoted as scenario C, and the region towards lower competition is dominated by senescence, denoted as scenario S. From Figure 3b, we see that Fisher information evolves in a non-monotonic manner, expressing dynamics dependent on which scenario we consider. Notably, Fisher information in the optimal case (scenario O) displays this same delay before the onset of cancer and before cell demographics reach a steady state. This transient feature is characteristic of our model and is also expressed by Fisher information. We now turn back to Fisher information in the context of statistical estimation.

## 6. Fisher Information and Statistical Inference

A key application of Fisher information in statistical estimation is the Cramér–Rao inequality. This states that if $\tilde{\lambda }$ is an unbiased estimator of λ, then the variance of the estimation is greater than or equal to the inverse of Fisher information:

(23) $$\langle {\left(\tilde{\lambda }-\lambda \right)}^{2}\rangle \geq \frac{1}{I(\lambda )}\;.$$

**Figure 4:**
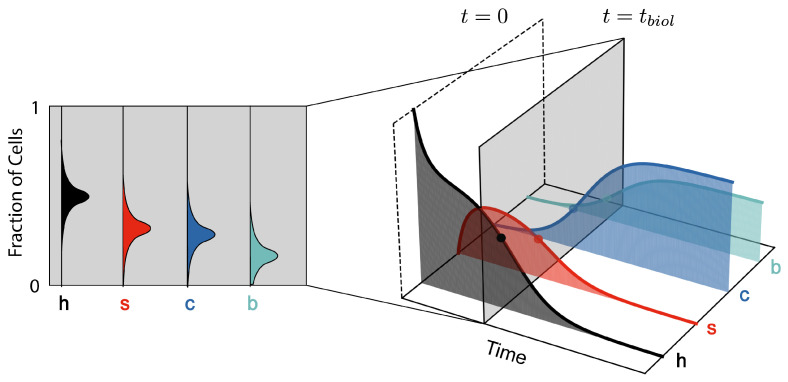
Fisher information parametrises the variance when inferring time $t_{biol}$ (**right**) from a sample of cells (**left**). This is analogous to estimating the true biological age of an organism from a sample of its cells, where biological and chronological time may differ.

More information about the parameter λ means a lower bound on the variance of the estimator $\tilde{\lambda }$, and thus higher precision. In our model, Fisher information quantifies how much information a sample of cell fractions $\rho_{i}=\left\{h,s,c,b\right\}$ provides about current time *t* (Figure 4). Figure 2b shows the time evolution of inverse Fisher information 1/I(t) for the four-state model. The evolution of 1/I(t) displays features which represent key phases in the model: initial accumulation of senescent cells, a transition from senescence to cancer, and late-stage proliferation of cancer at late times. At t=0, we have complete knowledge about the initial condition where all cells are healthy; thus, the inverse Fisher information vanishes. A detection of cancerous or senescent cells would indicate that cell populations have evolved beyond this initial state. Therefore, as senescent cells start to accumulate, the inverse Fisher information increases. A transient decrease in inverse Fisher information then follows during the transition from senescence- to cancer-domination, when the rate of population change is the greatest. According to Equation (23), this minimum in 1/I(t) suggests an optimal inference of *t*. Finally, inverse Fisher information diverges as cancer cells proliferate and, eventually, we reach a steady state between c and b cells. This can be explained by cell demographics reaching a steady-state (thus, zero information rate), and also by the inability to infer true time during the steady state based on a measurement (Fisher information and statistical inference).

## 7. Conclusions

Life, in its essence, is information. It is therefore unsurprising that ageing has been extensively studied through the lens of information theory [41,42,43,44,45,46]. For example, the “Information Theory of Ageing” posits that this loss of information is primarily in the form of epigenetic information critical for maintaining cellular function and identity [41,47]. Here, we have built upon a model of multicellular ageing based on the principle of optimal intercellular cooperation and competition [14,33] and shown that its dynamics can be understood in terms of Fisher information. Fisher information introduces an information-geometric interpretation which has been previously applied to replicator dynamics, natural selection, and the abstract Price equation [34,39,48], which we extended here to multicellular ageing. Identifying Fisher information allows us to consider inference and sampling via the Cramer–Rao inequality in the context of multicellular ageing; we are not suggesting any causal relationship. Conventionally, we understand the role of information in ageing by the principle that the disorder of the genome and epigenome only increases with age [46,49]. This “drift” can be used to estimate a metric of biological age, which may differ from an organism’s chronological age based on congenital conditions, lifestyle, and environment [50,51,52]. Lost information in our model relates to the ensemble of cell states composing a tissue, where uncertainty is inherent to statistical sampling, rather than damage to biological code. Our work provides new insights into the role of information in ageing, which could inspire potential strategies to extend healthy lifespans in multicellular organisms.

## Data Availability

No new data were created or analyzed in this study. Data sharing is not applicable to this article.

## Appendix A. Rewriting Master Equation as Effective Replicator Equation

In this appendix, we show that the master equation

(A1) $$\begin{array}{cc} \frac{d\rho_{i}}{dt}= & \left(f_{i}-\langle f\rangle \right)\;\rho_{i}+\sum_{j}\epsilon_{ij}\;\rho_{j}-\rho_{i} \end{array}$$

can be rewritten as an effective replicator equation with state-dependent replication rate

(A2) $$\begin{array}{cc} r_{i}= & f_{i}+\sum_{j}\epsilon_{ij}\;\frac{\rho_{j}}{\rho_{i}}=f_{i}+f_{i}^{\prime }\;. \end{array}$$

Indeed, the mutation part of Equation (A1) can be written as

(A3) $$\begin{array}{cc} \frac{d\rho_{i}}{dt} & =\sum_{j=1}^{n}\epsilon_{ij}\rho_{j}-\rho_{i}=\left(\sum_{j=1}^{n}\epsilon_{ij}\frac{\rho_{j}}{\rho_{i}}-1\right)\;\rho_{i}=\left(f_{i}^{\prime }-1\right)\rho_{i}\;. \end{array}$$

Next, we note that

(A4) $$\begin{array}{cc} \langle f^{\prime }\rangle & =\sum_{i}\rho_{i}\sum_{j}\epsilon_{ij}\frac{\rho_{j}}{\rho_{i}}=\sum_{i}\sum_{j}\epsilon_{ij}\rho_{j}=\sum_{j}\rho_{j}\sum_{i}\epsilon_{ij}=1 \end{array}$$

since $\sum_{i}\epsilon_{ij}=1$ and $\sum_{j}\rho_{j}=1$ to conserve probability. This means that

(A5) $$\langle f^{\prime }\rangle =1$$

and therefore, Equation (A1) takes the form

(A6) $$\frac{d\rho_{i}}{dt}=\left(f_{i}-\langle f\rangle \right)\;\rho_{i}+\left(f_{i}^{\prime }-\langle f^{\prime }\rangle \right)\;\rho_{i}=\left(r_{i}-\langle r\rangle \right)\;\rho_{i}\;,$$

i.e., it is an effective replicator equation.

## Appendix B. Derivation of Lagrangian

In the following, we provide the mathematical details for showing that the master equation

(A7) $$\frac{d\rho_{i}}{dt}=\left(r_{i}-\langle r\rangle \right)\;\rho_{i}\;$$

can be derived from the following Lagrangian [34]:

(A8) $$L=\sum_{i}\left(\frac{{\dot{\rho_{i}}}^{2}}{\rho_{i}}+\rho_{i}{\left(r_{i}-\langle r\rangle \right)}^{2}\right)+\lambda \left(\sum_{i}\rho_{i}-1\right)\;,$$

where

(A9) $$\begin{array}{cc} r_{i}= & f_{i}+\sum_{j}\epsilon_{ij}\;\frac{\rho_{j}}{\rho_{i}}\; \end{array}$$

and λ is a Lagrange multiplier that ensures conservation of total probability $\sum_{i}\rho_{i}=1$. The associated Euler–Lagrange equations read

(A10) $$\frac{𝜕L}{𝜕\rho_{i}}-\frac{d}{dt}\left(\frac{𝜕L}{𝜕{\dot{\rho }}_{i}}\right)=0\;.$$

The derivatives of the Lagrangian in Equation (A8) are respectively

(A11) $$\begin{array}{cc} \frac{𝜕L}{𝜕\dot{\rho_{i}}} & =2\frac{\dot{\rho_{i}}}{\rho_{i}} \end{array}$$

(A12) $$\begin{array}{cc} \frac{d}{dt}\left(\frac{𝜕L}{𝜕{\dot{\rho }}_{i}}\right) & =2\left(\frac{\rho_{i}\;\ddot{\rho_{i}}-{\dot{\rho_{i}}}^{2}}{\rho_{i}^{2}}\right) \end{array}$$

(A13) $$\begin{array}{cc} \frac{𝜕L}{𝜕\rho_{i}}\; & =-\frac{{\dot{\rho_{i}}}^{2}}{\rho_{i}^{2}}+\frac{𝜕}{𝜕\rho_{i}}\left(\sum_{k}\rho_{k}\;{\left(r_{k}-\langle r\rangle \right)}^{2}\right)+\lambda \;, \end{array}$$

where the average 〈r〉 is

(A14) $$\langle r\rangle =\sum_{j}\rho_{j}\;r_{j}\;.$$

Now, the middle term in Equation (A13) is calculated as

(A15) $$\begin{array}{cc} \frac{𝜕}{𝜕\rho_{i}}\left(\sum_{k}\rho_{k}\;{\left(r_{k}-\langle r\rangle \right)}^{2}\right) & ={\left(r_{i}-\langle r\rangle \right)}^{2}+\sum_{k}2\;\rho_{k}\;\left(r_{k}-\langle r\rangle \right)\frac{𝜕(r_{k}-\langle r\rangle )}{𝜕\rho_{i}}\;. \end{array}$$

Using Equation (A14), we find

(A16) $$\begin{array}{cc} \frac{𝜕}{𝜕\rho_{i}}\left(r_{k}-\langle r\rangle \right) & =\frac{𝜕r_{k}}{𝜕\rho_{i}}-r_{i}-\sum_{j}\rho_{j}\;\frac{𝜕r_{j}}{𝜕\rho_{i}} \end{array}$$

and therefore,

(A17) $$\begin{array}{cc} \frac{𝜕L}{𝜕\rho_{i}}\; & =-\frac{{\dot{\rho_{i}}}^{2}}{\rho_{i}^{2}}+{\left(r_{i}-\langle r\rangle \right)}^{2}+2\sum_{k}\rho_{k}\;\left(r_{k}-\langle r\rangle \right)\left(\frac{𝜕r_{k}}{𝜕\rho_{i}}-r_{i}-\sum_{j}\rho_{j}\frac{𝜕r_{j}}{𝜕\rho_{i}}\right)+\lambda \;. \end{array}$$

Note that

(A18) $$\begin{array}{cc} \sum_{k=1}^{n}\rho_{k}\left(r_{k}-\langle r\rangle \right) & =\underset{=\langle r\rangle }{\underset{︸}{\sum_{k}\rho_{k}r_{k}}}-\langle r\rangle \underset{=1}{\underset{︸}{\sum_{k}\rho_{k}}}=\langle r\rangle -\langle r\rangle =0\;, \end{array}$$

where we used $\sum_{k}\rho_{k}=1$. Therefore, Equation (A17) simplifies to

(A19) $$\begin{array}{cc} \frac{𝜕L}{𝜕\rho_{i}}\; & =-\frac{{\dot{\rho_{i}}}^{2}}{\rho_{i}^{2}}+{\left(r_{i}-\langle r\rangle \right)}^{2}+2\sum_{k}\rho_{k}\;\left(r_{k}-\langle r\rangle \right)\;\frac{𝜕r_{k}}{𝜕\rho_{i}}+\lambda \;. \end{array}$$

Combining these results, we obtain the Euler–Lagrange equations as

(A20) $$\begin{array}{cc} 2\;{\ddot{\rho }}_{i} & =\frac{{\dot{\rho_{i}}}^{2}}{\rho_{i}}+\rho_{i}{\left(r_{i}-\langle r\rangle \right)}^{2}+2\sum_{k}\rho_{i}\;\rho_{k}\;\left(r_{k}-\langle r\rangle \right)\;\frac{𝜕r_{k}}{𝜕\rho_{i}}+\lambda \rho_{i}\;. \end{array}$$

We now consider the master equation in the form

(A21) $$\dot{\rho_{i}}=\left(r_{i}-\langle r\rangle \right)\rho_{i}$$

and differentiate both sides with respect to time to check for equivalence with the Euler–Lagrange equations (A20):

(A22) $$\begin{array}{cc} \ddot{\rho_{i}} & =\dot{\rho_{i}}\left(r_{i}-\langle r\rangle \right)+\rho_{i}\frac{d(r_{i}-\langle r\rangle )}{dt}=\frac{{\dot{\rho_{i}}}^{2}}{\rho_{i}}+\rho_{i}\frac{d(r_{i}-\langle r\rangle )}{dt}\;, \end{array}$$

where we use Equation (A21) to transform the first term. Using the chain rule for derivatives

(A23) $$\frac{d}{dt}=\sum_{k}{\dot{\rho }}_{k}\;\frac{𝜕}{𝜕\rho_{k}}$$

we arrive at

(A24) $$\begin{array}{cc} \frac{d(r_{i}-\langle r\rangle )}{dt} & =\sum_{k}{\dot{\rho }}_{k}\frac{𝜕(r_{i}-\langle r\rangle )}{𝜕\rho_{k}}=\sum_{k}\rho_{k}\left(r_{k}-\langle r\rangle \right)\left(\frac{𝜕r_{i}}{𝜕\rho_{k}}-r_{k}-\sum_{j}\rho_{j}\frac{𝜕r_{j}}{𝜕\rho_{k}}\right)\;. \end{array}$$

Thus,

(A25) $$\begin{array}{cc} \ddot{\rho_{i}} & =\frac{{\dot{\rho }}_{i}^{2}}{\rho_{i}}+\rho_{i}\;\sum_{k}\rho_{k}\;\left(r_{k}-\langle r\rangle \right)\left(\frac{𝜕r_{i}}{𝜕\rho_{k}}-r_{k}-\sum_{j}\rho_{j}\frac{𝜕r_{j}}{𝜕\rho_{k}}\right)\;. \end{array}$$

Note that

(A26) $$\begin{array}{cc} \sum_{k}\rho_{k}\;r_{k}\;\left(r_{k}-\langle r\rangle \right) & =\sum_{k}\rho_{k}\;{\left(r_{k}-\langle r\rangle \right)}^{2}-\langle r\rangle \underset{=0}{\underset{︸}{\sum_{k}\rho_{k}\;\left(r_{k}-\langle r\rangle \right)}}=\sum_{k}\rho_{k}\;{\left(r_{k}-\langle r\rangle \right)}^{2}\;, \end{array}$$

which yields

(A27) $$\begin{array}{cc} \ddot{\rho_{i}} & =\frac{{\dot{\rho_{i}}}^{2}}{\rho_{i}}+\rho_{i}\;\sum_{k}\rho_{k}\;\left(r_{k}-\langle r\rangle \right)\left(\frac{𝜕r_{i}}{𝜕\rho_{k}}-\sum_{j}\rho_{j}\frac{𝜕r_{j}}{𝜕\rho_{k}}\right)-\rho_{i}\sum_{k}\rho_{k}\;{\left(r_{k}-\langle r\rangle \right)}^{2}\;. \end{array}$$

Comparing Equations (A20) and (A27) shows that the Euler–Lagrange equation and replicator equation will match if

(A28) $$\lambda =-2\sum_{k}\rho_{k}\;{\left(f_{k}-\langle f\rangle \right)}^{2}\;.$$
